# New Records of *Phenacoccus solenopsis* Natural Enemies in Europe and Taxonomic Additions on *Anagyrus matritensis*

**DOI:** 10.3390/insects16020169

**Published:** 2025-02-05

**Authors:** Michele Ricupero, Emanuele Porcu, Agatino Russo, Lucia Zappalà, Gaetano Siscaro

**Affiliations:** 1 Department of Agriculture, Food and Environment, University of Catania, 95123 Catania, Italy; agatino.russo@unict.it (A.R.); lucia.zappala@unict.it (L.Z.); gaetano.siscaro@unict.it (G.S.); 2 Department of Agriculture, University of Reggio Calabria, Feo di Vito, 89122 Reggio Calabria, Italy; emanuele.porcu@unirc.it

**Keywords:** *Anagyrus*, *Aenasius arizonensis*, barcoding, biocontrol, morphological identification, parasitoid, cotton mealybug, tomato

## Abstract

The cotton mealybug *Phenacoccus solenopsis* (Hemiptera: Pseudococcidae) is a polyphagous invasive species native to America and considered one of the major cotton pests in Asia. It is currently threatening horticultural and ornamental protected crops in Mediterranean countries. Due to ecological and environmental concerns, the conventional chemical control of *P. solenopsis* in new areas of introduction is being replaced by exploring the potential of indigenous natural enemies as a sustainable biological control tool. After *P. solenopsis* introduction in Sicily (Italy), field surveys were conducted on native natural enemies attacking the mealybug to select promising biocontrol agents for field applications. For the first time, *Aenasius arizonensis* (Hymenoptera: Encyrtidae) was reported in Europe, and the native *Anagyrus matritensis* (Hymenoptera: Encyrtidae) was recorded in association with *P. solenopsis*. The two parasitoid species were identified by morphological features and molecularly using a portion of the mitochondrial cytochrome oxidase subunit I (mtCOI) gene. Because of missing information, additional morphological features were provided for the morphological identification of *A. matritensis*. In addition, the generalist predators *Cryptolaemus montrouzieri*, *Hippodamia variegata* and *Parexochomus nigripennis* (Coleoptera: Coccinellidae) were also recorded attacking the invasive mealybug.

## Simple Summary

*Phenacoccus solenopsis* (Hemiptera: Pseudococcidae) is threatening protected horticultural and ornamental crops in Mediterranean countries, and research into indigenous natural enemies is needed as a sustainable biological control tool. Field surveys were carried out to discover the natural enemies attacking the cotton mealybug in Sicily (Italy) where the pest is currently present. Hymenopteran parasitoids and generalist coccinellid predators were reported. *Aenasius arizonensis* (Hymenoptera: Encyrtidae) was reported for the first time in Europe, and *Anagyrus matritensis* (Hymenoptera: Encyrtidae) was recorded for the first time in association with *P. solenopsis*. The two parasitoid species were identified by morphological features and molecular tools using a partial sequence of the COI mitochondrial gene. Further morphological details were also added to the original description for *A. matritensis*. The generalist predators *Cryptolaemus montrouzieri*, *Hippodamia variegata* and *Parexochomus nigripennis* (Coleoptera: Coccinellidae) were also recorded preying on the cotton mealybug.

## 1. Introduction

The unintentional introduction of invasive exotic insect species is an increasing phenomenon worldwide that is exacerbated by climate change and leads to an adverse impact on crop production [1]. After the dispersal and expansion of the geographical distribution of such organisms, new trophic associations can occur [2]. Among recent cases reported in the Mediterranean, noteworthy is the introduction of the cotton mealybug *Phenacoccus solenopsis* Tinsley (Hemiptera: Pseudococcidae). This invasive species is native to North America; it is currently present in all continents, and it is considered one of the key pests of cotton in Asia [3]. Within the Palaearctic region, the species has been reported on during the last ten years in several countries in the Mediterranean basin [4,5]. Although in these regions, *P. solenopsis* has been primarily recorded on wild hosts and ornamentals (e.g., *Hibiscus* sp. and *Lantana* sp.), in Israel and Egypt, the mealybug has become a serious pest in cotton fields and solanaceous protected crops, namely in bell pepper and tomato greenhouses [6].

*P. solenopsis* is a highly polyphagous sap-sucking pest that can feed on over 200 host plants belonging to about 60 botanical families. This mealybug feeds on phloem sap mainly on the aboveground plant parts and produces honeydew and waxy secretion. It is known to have high fecundity (from 150 to 600 eggs per female), and it can complete up to 15 overlapping generations under field conditions. Its feeding activity also causes chlorosis and can induce plant growth reduction and even plant death [3]. Although the honeydew emitted by the mealybug is not abundant, *P. solenopsis* establishes mutualistic relationships with several species of ants (Hymenoptera: Formicidae) including the invasive red imported fire ant *Solenopsis invicta* Buren [7]. This aspect could be detrimental for agricultural production and trade in areas where both species are present [4,8].

As the introduction of *P. solenopsis* raises ecological and environmental concerns also due to conventional chemical control [3,9], there is a growing interest in exploring the potential of indigenous natural enemies as sustainable control tools. Biological control using natural enemies, particularly parasitoids, has been thought to be the key factor in suppressing *P. solenopsis* populations in agricultural systems. More than twenty-five records of parasitoid species, mainly belonging to the hymenopteran family Encyrtidae, are reported as parasitizing on *P. solenopsis* [10]. A recent study recorded a total of 18 hymenopteran parasitoids belonging to 11 genera and 4 families in Southern China. Eight of these species are primary parasitoids, with *Aenasius arizonensis* (Girault) (Hymenoptera: Encyrtidae) being the dominant taxon, while the remaining ten species are most likely hyperparasitoids [11].

A further evaluation and use of parasitoid wasps in biological control programmes against *P. solenopsis* require accurate species identification in new invaded areas [3]. However, given the diversity of parasitoid wasps associated with *P. solenopsis*, species identification via morphology can be difficult. This aspect is particularly relevant for non-hymenopteran parasitoid specialists, as these organisms are typically small and morphologically similar, requiring a thorough examination of detailed morphological characters [12]. However, this limitation can be overcome by the DNA sequencing of mitochondrial cytochrome oxidase subunit I (mtCOI) since it has become an adequate tool for species identification in insects [13,14].

In this study, we used an integrated approach (morphological and molecular) to identify parasitoids associated with *P. solenopsis*, and we also recorded its predators for the first time in Italy. Moreover, since the morphological characterization of *Anagyrus matritensis* (Mercet) (Hymenoptera: Encyrtidae) was lacking information, we provided additional morphological details and molecular identifications of this species. The findings of this study may help to identify the natural enemies of *P. solenopsis*, as this is an important step towards the use of natural enemies as biological control agents against this serious insect pest in Mediterranean agroecosystems.

## 2. Materials and Methods

### 2.1. Insect Sampling

Between October 2023 and November 2024, we conducted periodic samplings of *P. solenopsis* from infested protected crops and ornamental gardens in Sicily, Italy (Table 1). Specifically, we inspected the aboveground parts of infested plants (both cultivated and wild) in four sites located in the southern coast of the island. We collected female mealybugs using a soft paintbrush and stored them in ventilated boxes for laboratory identification. Each sample was placed in a plastic bag and taken to the laboratory for examination. The colonies were maintained in an aerated plastic box in a climate cabinet (25 ± 1 °C and 60 ± 5% RU), and each parasitized mealybug specimen was singularly isolated in small vials until the emergence of adults. The newly emerged parasitoids were initially preserved in 80% alcohol. These were card-mounted, and at least one specimen of each species (or parts of one specimen) was mounted on slides.

The terminology used for the description refers to Noyes and Hayat [12]. All measurements and pictures reported were obtained using a digital image acquisition and processing system connected to a microscope; observations were made using a Leica Flexacam C3 video camera connected to a Zeiss Axiophot optical microscope. Identification was achieved through an initial morphological study. To this aim, the authors used the most relevant identification keys and literature sources [12,15,16,17,18]. Parasitoid specimens were also identified by molecular analyses on mitochondrial DNA sequences as described below.

### 2.2. Molecular Identification

The molecular identification of the collected parasitoids was performed by sequencing the amplified mtCOI gene fragment as follows. DNA was isolated from a single specimen of *A. arizonensis* and *A. matritensis* from each site using the E.Z.N.A.^®^ Tissue DNA Kit (Omega Bio-Tek, Inc., Norcross, GA, USA). A non-destructive DNA extraction protocol was followed to allow for the morphological identification of the processed specimens. Samples of *Anagyrus vladimiri* Triapitsyn (Hymenoptera: Encyrtidae) as a positive DNA control and a negative control without DNA were also included in the extraction. Universal primer pairs LCO1490 (5′-GGTCAACAAATCATAAAGATATTGG-3′), HCO2198 (5′-TAAACTTCAGGGTGACCAAAAAATCA-3′), C1-J-2195 (alias Jerry, 5′-TTGATTTTTGGTCATCCAGAAG-3′) and TL2-N-3014 (alias Pat, 5′-TCCAATGCACTAATCTGCCATATTA-3′) were used to amplify the expected ≈630 bp and ≈800 bp of the mtCOI target region for *A. arizonensis* [13] and *A. matritensis* [19], respectively. A PCR was performed according to the protocol proposed by [4]. Reactions were performed in 20 μL volumes with 0.85X FailSafe^TM^ PCR 2X PreMix F (Lucigen Corporation, Middleton, WI, USA), 0.5 μM of each primer 10 μM, 1.5 U Taq DNA Polymerase 5U (Invitrogen, Thermo Fisher Scientific, Waltham, MA, USA) and 2 μL DNA template. Cycling conditions were as follows: 96 °C for 5 min, 35 cycles at 96 °C for 45 s, 45 °C for 1 min and 72 °C for 1 min, followed by a final cycle at 72 °C for 10 min. Reactions and cycling conditions were carried out in an Applied Biosystems^TM^ MiniAmp^TM^ Plus Thermal Cycler (Waltham, MA, USA). PCR products were first checked by electrophoresis using a 1% agarose gel, and those of the expected size were shipped to BMR Genomics (Padova, Italy) for purification and sequencing using Sanger’s method. The coding regions were manually checked for errors and trimmed for low quality in Unipro UGENE version 1.26.1. The resulting FASTA files were aligned to reference sequences from the National Center for Biotechnology Information (NCBI) GenBank^®^ using the Basic Local Alignment Search Tool (BLAST) sequence analysis tool for species identification. All sequences were deposited in GenBank under the following accession numbers: PQ736881 for *A. arizonensis* and PQ736880 for *A. matritensis*.

## 3. Results

### 3.1. Natural Enemies

**Figure 1:**
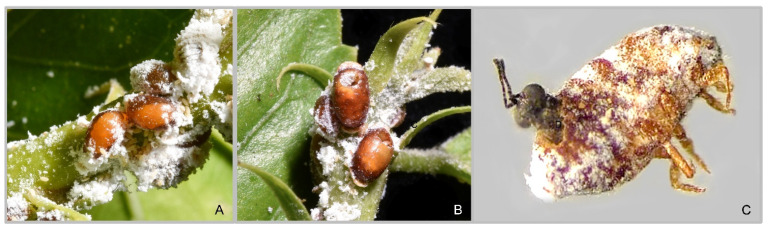
*Phenacoccus solenopsis* parasitized cocoons with *Aenasius arizonensis* pupae visible in transparency (**A**), *Phenacoccus solenopsis* cocoons with *Aenasius arizonensis* emerging holes (**B**), *Anagyrus matritensis* adult emerging from *Phenacoccus solenopsis* cocoon (**C**).

Among the surveyed sites in Sicily (Table 1), we recorded the exotic parasitoid *Aenasius arizonensis* on *P. solenopsis*-infested ornamental plants located in Catania and Siracusa (Figure 1A,B), while the native *Anagyrus matritensis* (Figure 1C) was only found in Catania. In tomato crops and urban ornamentals located in Marina di Ragusa and Catania, the generalist predators *Cryptolaemus montrouzieri* (Mulsant), *Hippodamia variegata* (Goeze) and *Parexochomus nigripennis* (Erichson) (Coleoptera: Coccinellidae) were often reported in association with *P. solenopsis* infestation (Table 1).

### 3.2. Materials Examined

Specimens were collected in the samples taken from the field; the further materials examined came from a laboratory rearing.

Depository: the examined specimens were deposited at the Department of Agriculture, Food and Environment of the University of Catania (Italy). Other specimens were also deposited as follows: *A. arizonensis* (Girault) (Hymenoptera: Encyrtidae), 10 ♀♀ 7 ♂♂ Catania, Italy 2 October 2024 ex *P. solenopsis* Tinsley (Hemiptera: Pseudococcidae) on *H. rosa-sinensis* L. at The Smithsonian Institution, National Museum of Natural History Washington, DC, USA.

Abbreviations:

AOL—the minimum distance between the posterior and anterior ocellus.
OOL—the minimum distance between the eye margin and the nearest posterior ocellus.
POL—the minimum distance between the posterior ocelli.
F_1–6_—the antennal articles.

***Aenasius arizonensis* (**Girault**)**

*Aenasius bambawalei* Hayat 2009 syn.nov.

The species was originally described in detail by Hayat [17], and additional descriptions were provided later by other authors [20,21,22,23]. Some featured morphological characters are reported below.

Distribution: China, India, Iran, Iraq, Israel, Pakistan, Turkey and USA [6,10,12,24,25]. This is the first report of the species and the genus in Italy and Europe.

**Figure 2:**
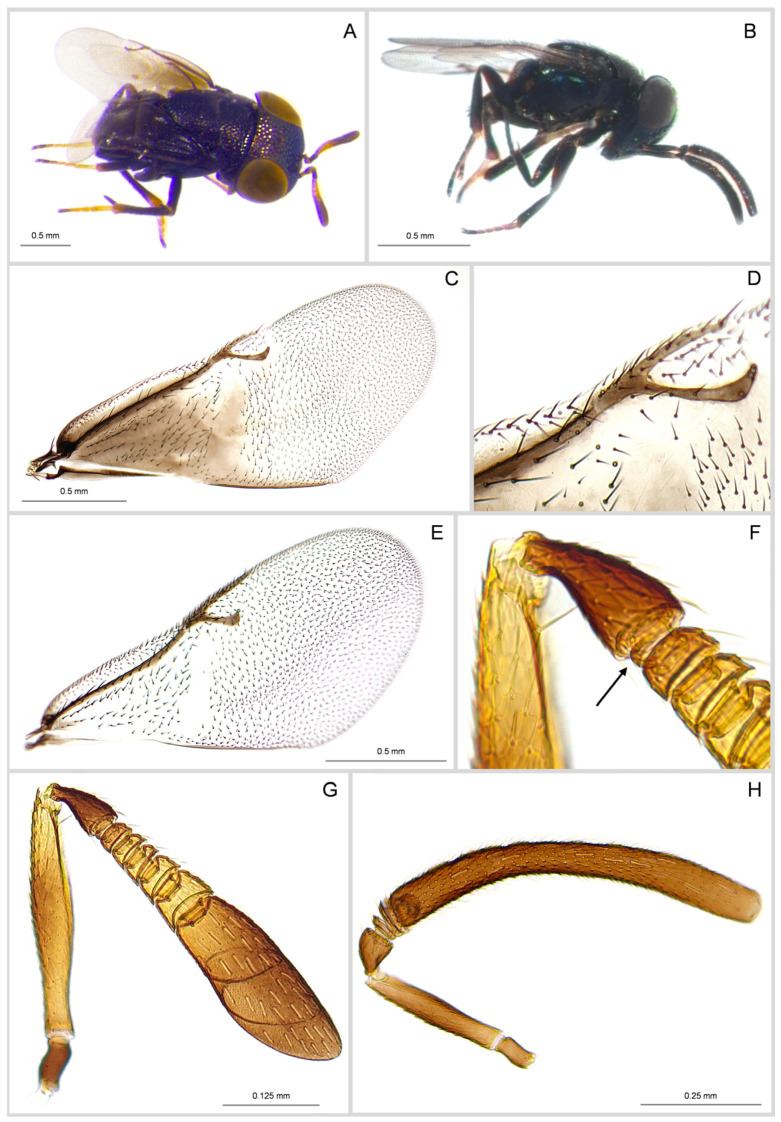
*Aenasius arizonensis* (Girault), female, habitus in dorso-lateral view (**A**); male, habitus in lateral view (**B**); female, fore wing (**C**); female, fore wing distal venations (**D**); male, fore wing (**E**); female antenna, particular of pedicel and first funicle segments, arrow indicates ring-shaped first segment (**F**); female antenna (**G**); male antenna (**H**).

**Female**. Length: 1.50–2.30 mm. Body shiny black with metallic reflections bluish green to blackish on thorax and bronzy-violet to bluish green on abdomen (Figure 2A).

Antenna (Figure 2G) with scape cylindrical, yellowish brown, with a brownish patch in the middle; pedicel black, segments F_1_, F_2_, F_3_ brownish and F_4_, F_5_ and F_6_ yellowish brown; clava dark brown, basally brownish yellow, strongly truncate at apex. Antennal funicle 6-segmented; segment F_1_ is very narrow and poorly visible in the specimens that were collected in Sicily (Figure 2F). Fore wing (Figure 2C) brownish basally with distal half and costal cell hyaline; hind wing hyaline; the postmarginal vein slightly shorter than stigmal one. Legs, including coxae, black with apical portion of tibiae yellowish brown; all tarsi yellowish to yellowish brown and mid-tibial spur brown. Gaster smooth, very finely reticulated, shorter than thorax (thorax is 1.25× the abdomen length).

**Male**. Length: 1.10–1.50 mm. It is generally smaller than female (Figure 2B), and the sculpture and the colour of mesothoracic dorsum are similar to those of the female. Fore wing hyaline with the postmarginal vein longer than stigmal one (Figure 2E); hind wings hyaline. Antennal funicle with only two anelli and clava with a single, very long article (Figure 2H).

***Anagyrus matritensis* (**Mercet**)**

*Gyranusa matritensis* Mercet, 1921.

*Blastothrix orbitalis* Ruschka, 1923. Synonymy by Koponen and Askew [26].

*Anagyrus orbitalis* (Ruschka): Trjapitzin, 1972: 255. Synonymy by Koponen and Askew, 2002 [24].

*Anagyrus matritensis* (Mercet): Noyes, 1981 [27].

The species was originally described by Mercet [28] on the basis of four females and five males collected in 1917 in the province of Madrid on a *Pinus halepensis* plant infested by an unidentified armoured scale; however, the scale has not been reported as a natural host. In this paper, only a small drawing of the female antenna is reported.

Distribution: Palaearctic with presence recorded in several European and Asian countries [25]. This is the first report of *A. matritensis* in Italy and its first record on *P. solenopsis*.

*Blastothrix orbitalis* was described by Ruschka [29] from four females collected in Austria in 1915 and two males from Mayr’s collection; no information is given on the natural host.

The latest morphological description of *A. matritensis* was published in 2014 on two females caught in a yellow panel trap in Shandong Province, China [30]. The natural host and the male are unknown to the authors.

Other references on *A. matritensis* include morphological comparisons with other species, as follows:

(i) Compared with *A. descriptus* sp. n.; distinguishing characters for *A. matritensis* are not fully consistent with the original description; no details on males or host [31].
(ii) Compared with *A. kilinceri* sp. n.; distinguishing characters are not fully consistent with the original descriptions; no details on males or host [32].
(iii) The morphological details of *A. matritensis* are given in an identification key of the *Anagyrus* parasitoids of *Planococcus* and *Pseudococcus* mealybugs in eastern Spain; female habitus and fore wing are illustrated. Although the parasitoid is considered to be common in Spanish citrus orchards, information on the specific natural host was not provided [33].

Pending the complete review of this large genus-group of parasitoids, the identification of this species was confirmed by Dr. Emilio Guerrieri CNR-IPSP, Italy. The emergence of *A. matritensis* from *P. solenopsis* represents, to the best of our knowledge, a new association and opens new opportunities for the biological control of this pest in crop systems. The biology of this species is largely unknown, and studies of its host preference and parasitism behaviour are underway. The typical series of this species has not been studied, but the specimens collected are consistent with the original description [28] and other recent morphological descriptions of this species [30,33]. However, some additional characters and relevant variations found in our samples are included in the following description.

**Figure 3:**
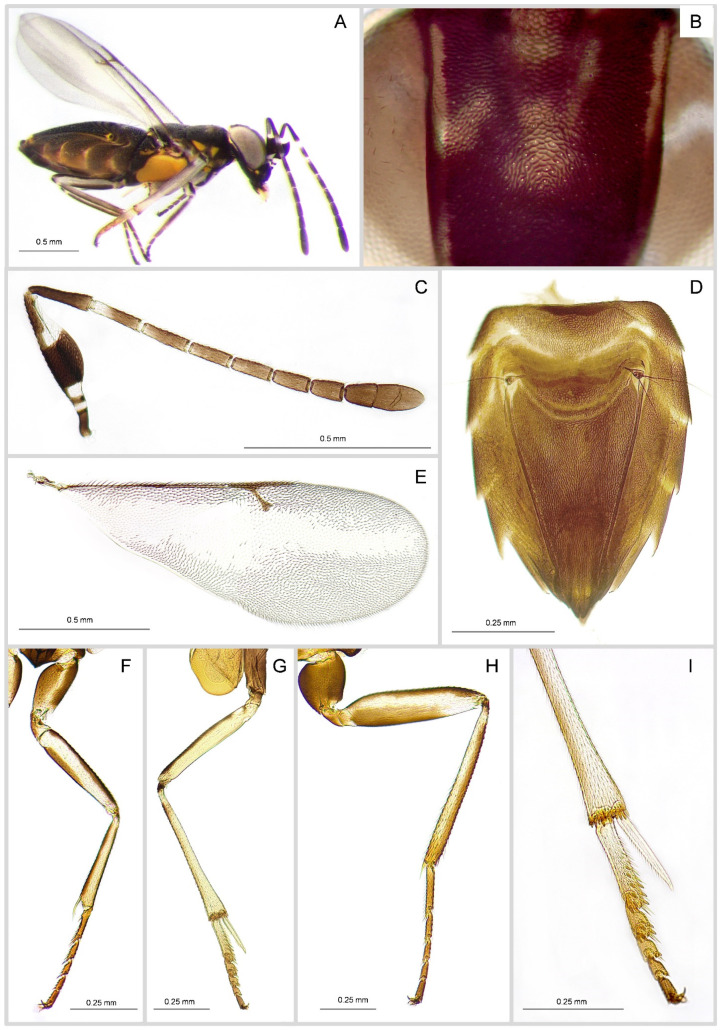
*Anagyrus matritensis* (Mercet) female. (**A**) Habitus in lateral view; (**B**) frontovertex with detail of sculpture; (**C**) antenna; (**D**) gaster; (**E**) fore wing; (**F**) fore leg; (**G**) mid-leg; (**H**) hind leg; (**I**) mid-tibial spur and tarsus.

**Female**. Length 1.6–2.4 mm. Body slightly slender and not dorsoventrally flattened (Figure 3A); head testaceous brown with eye borders and occiput yellowish; a small yellowish spot is visible at the lower margin of the eye; antenna blackish; the base and the apical half of the scape and the basal part of F1 are whitish; the white proportion in the first antennal segment varies from one-fifths to three-fifths of the whole segment even though in the specimens examined, the white portion never exceed one-quarter; pronotum with lateral posterior margin and small light yellow spot on each side, prepectus and mesopleuron yellow; tegulae whitish with brown spots basally and apically; fore wing hyaline with a pattern of dark and pale setae forming a broad longitudinal hyaline band from the basal extreme to the apical one; hind wing entirely hyaline; legs whitish, with brown coxae and lines running through front and back on femurs and tibiae, particularly noticeable in the fore and hind legs (Figure 3F–H).

Frontovertex, pronotum, mesoscutum, scutellum, propodeum laterally and gaster, with elongate to striate sculpture (Figure 3B,D); scape mostly reticulate; setae on frontovertex as long as diameter of anterior ocellus; head, mesosoma and gaster with dense white bristles.

Head in frontal view slightly (1.04×) wider than high; frontovertex 0.4× as broad as head; gena as long as the distance from antennal toruli; OOL larger than the diameter of one ocellus; ocelli forming an acute angle of about 85°; AOL = POL; eyes more than 2.5× as long as malar space; inner eye margins slightly diverging ventrally; dorsal margin of antennal torulus slightly above ventral eye margin; torulus separated from mouth margin by a little less than half its height; antennal scrobes moderately deep, not meeting apically; malar sulcus distinct. Mandible with two acute teeth; palp formula 4:3.

Antenna with scape elongate 3.7× longer than broad and about 2× as long as the pedicel (Figure 3C); the latter slightly longer than F_1_; succeeding articles gradually slightly smaller; clava somewhat longer than previous two articles joined. Relative measurements: scape 986; pedicel 472; F_1_ 383; F_2_ 357; F_3_ 313; F_4_ 292; F_5_ 263; F_6_ 221; clava 607.

Mesoscutum barely as long as scutellum; posterior margin of pronotum concave; propodeum greatly irregular longitudinally with middle part very narrow; pleura reticulate-squamous; scutellum 0.9× as long as wide.

Fore wings with a hyaline longitudinal band running through the centre of the disc from the base to the apex; costal cell visible only in proximal third; marginal setae very short; fore wing 2.8× as long as broad (Figure 3E); linea calva partially visible and interrupted posteriorly; marginal and postmarginal veins combined are 2.2 × the stigma vein; postmarginal vein slightly longer than marginal vein and 1.4× as long as stigmal vein; hind wing completely hyaline and 4.3× as long as broad.

Distal end of tibia with a row of 11 thick and blunt spines; mid-tibial spur nearly as long as metatarsal (Figure 3I); apical half of metatarsus with a double row of five thick, blunt spines; second article with two apical spines; third article with only one blunt apical spine; fourth article without spines.

Gaster 1.7× longer than mesosoma (Figure 3D), narrowed towards the apex; dorsal surface of segments finely reticulated. Cercal plate is positioned at the upper quarter of the gaster, cercal bristles long and blackish in colour. Ovipositor short and not exserted, about one-third as long as the gaster.

**Figure 4:**
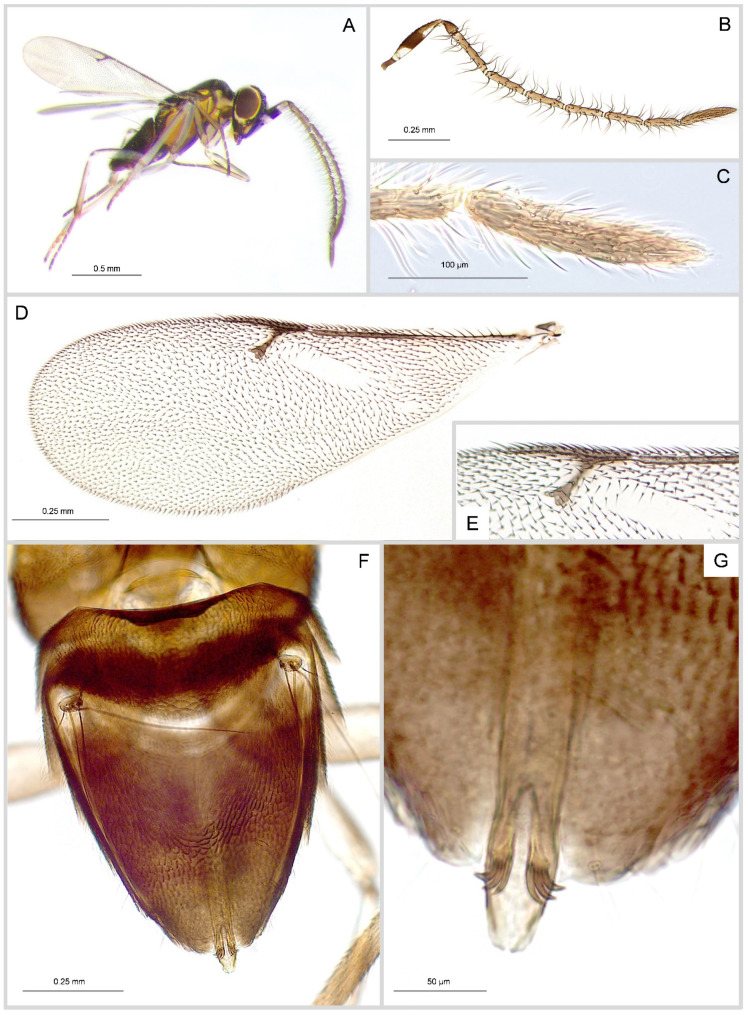
*Anagyrus matritensis* (Mercet) male. Habitus in lateral view (**A**); antenna (**B**); club showing scale-like sensilla (**C**); fore wing (**D**); fore wing distal venations (**E**); gaster (**F**); genitalia (**G**).

**Male**. Length: 0.9–1.4 mm. General colouration as in female (Figure 4A), mostly testaceous and yellowish except for the head, the dorsal part of the mesosoma and partly the gaster, which are brownish. A small yellow spot is visible on each side of the base of the antennae; a yellow band is visible around the eye; legs are whitish, with brownish middle and hind coxae and lines running anteriorly and posteriorly through the femurs and tibiae, particularly pronounced on the fore and hind legs; the base of the middle tibiae has a whitish ring.

Antenna (Figure 4B) with the scape brownish and white at the base and at apex; clear-brownish funicle; scape elongated 4.4× long as broad and 4× the pedicel; all funicle segments are much longer than broad with F_1_ the longest and F_6_ the shortest; clava 1.7 longer than F_6_ with scale-like sensilla in the basal half (Figure 4C); scattered long setae on funicle, the longest being more than three time as long as the segment width. Relative measurements: scape 612, pedicel 153, F_1_ 484, F_2_ 411, F_3_ 401, F_4_ 366, F_5_ 377, F_6_ 330, clava 584.

Wings entirely hyaline with blurred hyaline fringe; fore wing 2.7× as long as broad (Figure 4D); hind wing 4.1× as long as broad; linea calva well defined and interrupted by five rows of setae; postmarginal vein 2× longer than marginal vein and 1.5× the stigma vein (Figure 4E); marginal and postmarginal veins combined are 2.3× the stigma vein.

Gaster almost triangular (Figure 4F) about 1.2× as long as broad (at its base in dorsal view); cercal plate positioned at the upper quarter of the gaster, cercal bristles long (the longest about half the gaster length) and blackish; genitalia about 2× as long as mid-tibia; phallobase with well-developed digit, each with three short hooks (Figure 4G).

### 3.3. Molecular Identification

Based on mtCOI sequences, each parasitoid species that emerged from the *P. solenopsis* individuals sampled in geographically separated areas in Sicily shared the same haplotype. Subsequently, we confirmed the morphological identification of *A. arizonensis* through mtCOI fragment amplification from genomic DNA, followed by direct sequencing and BLAST searches. The resulting sequences were aligned to reference sequences from NCBI compared with publicly available data on GenBank and yielded an identity score of 100% and E-value = 0.0 with an *A. arizonensis* isolate from Southern China (accession number MT775615.1). On the other hand, no publicly available sequences for *A. matritensis* targeting the ≈800 mtCOI region were previously deposited on GenBank; therefore, our submission can be considered the first one for this taxon.

## 4. Discussion

Among alien invasive species, the solenopsis mealybug can cause a series of negative implications for the sustainability of cultivation systems in the Mediterranean because of its cryptic behaviour and the number of hosts [3]. For these reasons, the exploitation of sustainable control tools such as biological control are needed for mealybug pest control, and the quick identification of indigenous natural enemies is necessary to provide effective measures [10]. Following the introduction of *P. solenopsis* in Sicily [4], we reported for the first time in Europe the exotic parasitoid *A. arizonensis*, and we also recorded in Italy the presence of *A. matritensis* on *P. solenopsis* using morphological and molecular identification tools. In addition, three coccinellid predators (Coleoptera: Coccinellidae), namely *C. montrouzieri*, *H. variegata* and *P. nigripennis*, were consistently reported attacking *P. solenopsis* in different sites in the southern coast of the island.

The solitary endoparasitoid *A. arizonensis* was reported attacking *P. solenopsis* in India, and it has been documented as a very effective biological control agent with a parasitization rate up to 72% on cotton fields [20]. However, a hyperparasitoid that attacks *A. arizonensis*, *Promuscidea unfasciativentris*, may potentially reduce the efficiency of this biological control agent [10]. The discovery that the *A. arizonensis* haplotype from Sicily matches with the haplotype from individuals isolated in China suggests a possible introduction from Asia as we previously documented for *P. solenopsis* [4]. The introduction of exotic natural enemies by their hosts is likely to occur in the case of cryptic species such as mealybugs. Moreover, since we found *A. arizonensis* in the urban centre, this suggests a clear phenotypic plasticity of the species to adapt to a warmer and polluted environment with potential positive consequences for its biocontrol activity [34]. Future works should be promoted on the distribution, field parasitism rates and biology of *A. arizonensis* in regions of recent introduction, following the approach used for other invasive exotic pests [35,36].

The genus *Anagyrus* includes over 350 described species of primary endoparasitoids of mealybugs. Many of these species have been used in biological control programmes against mealybug pests [19]. Various recent revisions [19,37,38] pointed out and separated entities misidentified for a long time (e.g., the *Anagyrus pseudococci* species complex). Several species within this complex have been included in biocontrol programmes [19], and in this context, the correct identification of pests and released biological control agents is even more crucial for potential environmental and applied consequences. For this purpose, the combination, as performed in this study, of morphological and genetic approaches is essential [39]. To the best of our knowledge, this is the first record of *A. matritensis* in Italy, and more interestingly, this is the first confirmed report of a natural host for this parasitoid. The species is considered to be native to the Mediterranean area, and most likely, it switched to *P. solenopsis* from native mealybugs hosts which are still unknown and should be further investigated. Species identification is crucial when biological control strategies are implemented [40]. The accurate identification of natural enemies is crucial in rearing facilities, with the objective of targeting the cotton mealybug and thereby preventing potential economic losses.

More than 50 natural enemies, including both predators and parasitoids, have been recorded attacking *P. solenopsis* across its wide distribution range, with coccinellid predators (Coleoptera: Coccinellidae) being the most important taxon [10]. We consistently found the coccinellids *C. montrouzieri*, *H. variegata* and *P. nigripennis* to be in association with *P. solenopsis*. *Cryptolaemus montrouzieri* was often reported preying on *P. solenopsis* in China, India, Israel, Pakistan and Australia [6,10], and its role can be exploited in integrated pest management programmes against mealybug pests [41]. Meanwhile, *H. variegata* and *P. nigripennis* have been reported to feed on *P. solenopsis* in India [10] and Israel [6], respectively, and their function as promising biological control agents should be studied in laboratory and field conditions. Moreover, considering that the abundance of generalist natural enemy trophic groups leads to a significant reduction in pest abundance, understanding how plant diversity associated with tomato field structure arthropod food webs may improve the ecological management of *P. solenopsis* [42,43].

The integrated (morphological and molecular) taxonomic approach used in this study to investigate the natural enemy community associated with *P. solenopsis* should improve the accurate identification of these organisms, which is of great importance for the development of an effective biological control programme for this invasive pest in the Mediterranean. In addition, this study contributes to a better understanding of some of the ecological interactions between the invasive *P. solenopsis* and the natural enemies associated with it. Overall, continuously updating the morphological taxonomic keys associated with new molecular tools for the fast and precise identification of invasive pests is warranted.

## Figures and Tables

**Table 1:** Location, sites and host plants where natural enemies were found in association with *Phenacoccus solenopsis* in Sicily between October 2023 and November 2024.

Location	Coordinates		Host Plant	Species
	Latitude (N)	Longitude (E)		
Marina di Ragusa	36°47′11.7″	14°34′21.1″	*Lycopersicon esculentum* Mill. (Solanaceae) *Hibiscus* sp. L. (Malvaceae)	*Cryptolaemus montrouzieri* *Parexochomus nigripennis*
Siracusa	36°58′34.8″	15°13′55.5″	*Hibiscus* sp. L. (Malvaceae)	*Aenasius arizonensis*
Catania	37°30′42.7″	15°04′55.2″	*Hibiscus* sp. L. (Malvaceae)	*Aenasius arizonensis*
	37°31′35″	15°04′15.9″	*Lantana camara* L. (Verbenaceae)	*Anagyrus matritensis* *Cryptolaemus montrouzieri* *Hippodamia variegata* *Parexochomus nigripennis*

## Data Availability

The datasets generated in the study are available from the corresponding author upon request.
